# An Intravesical *Akkermansia muciniphila*‐Based Chemo‐Immunotherapeutic Platform for Bladder Cancer

**DOI:** 10.1002/advs.77241

**Published:** 2026-08-17

**Authors:** Rongkang Li, Anguo Zhao, Qi Zhuang, Lei Peng, Rui Liang, Jiadong Zhao, Yang Liu, Dashi Deng, Peng Yu, Xiqi Peng, Shaohua Zhang, Song Wu

**Affiliations:** ^1^ Department of Urology The Second Hospital & Clinical Medical School Lanzhou University Lanzhou China; ^2^ Department of Urology South China Hospital Medical School Shenzhen University Shenzhen China; ^3^ Department of Urology The Fourth Affiliated Hospital of Soochow University Medical Center of Soochow University Suzhou Dushu Lake Hospital Suzhou China; ^4^ Guangdong Key Laboratory for Biomedical Measurements and Ultrasound Imaging National‐Regional Key Technology Engineering Laboratory for Medical Ultrasound School of Biomedical Engineering Shenzhen University Medical School Shenzhen China

**Keywords:** akkermansia muciniphila, bladder cancer, chemo‐immunotherapy, ferroptosis, intravesical therapy

## Abstract

Bladder cancer (BC) is a highly recurrent urinary malignancy for which current intravesical immunotherapy and chemotherapy are limited by insufficient efficacy, toxicity, excessive inflammation, and drug resistance. The probiotic *Akkermansia muciniphila* (AKK), known for its ability to modulate immune responses and suppress pathological inflammation, provides a promising immunomodulatory component to address these limitations. Here, an intravesical bacteria‐based chemo‐immunotherapeutic platform is developed by loading doxorubicin (DOX) onto pasteurized AKK modified with F127, yielding AKK‐F127/DOX (AF/D). Compared with free DOX, AF/D more effectively inhibits bladder cancer cell proliferation, migration, and invasion, while inducing apoptosis, ferroptosis, immunogenic cell death, and suppressing the NF‐κB inflammatory pathway. AF/D also promotes dendritic‐cell maturation, pro‐inflammatory cytokine secretion, M1‐like macrophage polarization, antigen cross‐presentation, and tumor‐specific CD8^+^ T‐cell priming. In orthotopic bladder cancer models, intravesical AF/D shows superior antitumor efficacy and survival benefits over single‐agent treatments, remodels the local immune microenvironment, enhances systemic antigen‐specific CD8^+^ T‐cell responses in the spleen, and maintains a favorable safety profile. These findings establish AF/D as a promising intravesical chemo‐immunotherapeutic strategy for bladder cancer.

## Graphical abstract

Pasteurized *Akkermansia muciniphila* is engineered into an intravesical F127/doxorubicin platform that couples localized chemotherapy with immune remodeling. By inducing apoptosis, ferroptosis, and immunogenic cell death, this chemo‐immunotherapeutic system enhances dendritic‐cell maturation, antigen cross‐presentation, and tumor‐specific CD8^+^ T‐cell responses, achieving potent bladder tumor suppression with favorable tolerability.

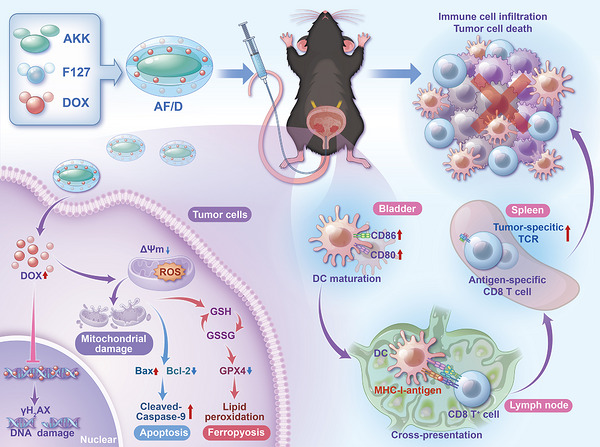

## 1 Introduction

Bladder cancer (BC) is one of the most common malignant tumors in the urinary system, characterized by a high recurrence rate, which often requires long‐term management and treatment [1, 2]. Globally, BC is ranked as the ninth most common cancer, and its treatment and follow‐up management are challenging; the associated healthcare costs impose a significant burden on both patients and healthcare systems, creating considerable challenges [3, 4]. Clinical treatment for BC mainly includes surgery, chemotherapy, and immunotherapy, but monotherapies often show limited efficacy, leading to tumor recurrence and the potential for severe side effects [5]. Intravesical Bacillus Calmette‐Guérin (BCG) is a cornerstone immunotherapy in bladder cancer management, yet 30%–40% of treated patients still experience recurrence or progression, and local or systemic toxicities may limit tolerability and lead to discontinuation [1, 6, 7]. In addition, postoperative intravesical chemotherapy is also widely used [8]. However, the efficacy is often weaker than BCG, and resistance remains a significant issue; moreover, intravesical chemotherapy often induces local irritative toxicity and rarely generates durable antitumor immune memory, collectively limiting compliance and recurrence control [9, 10]. Therefore, combining immunotherapy and chemotherapy represents a promising direction in bladder cancer treatment, as it improves therapeutic efficacy while reducing the drug burden and maintaining patient safety. A comprehensive strategy that integrates chemotherapy and immunotherapy can better leverage their complementary advantages, thereby reducing recurrence risk and optimizing treatment outcomes.

At the same time, the difficulty in bladder cancer treatment largely stems from inadequate regulation of the tumor's local immune microenvironment [11, 12]. Ideal treatment strategies aim to reshape the tumor microenvironment from an “immune‐suppressive/inflammatory dysregulated” state to an “immune‐activated/inflammation‐controlled” state, enhancing therapeutic sensitivity while protecting normal tissues [13, 14]. Chronic inflammation has increasingly been recognized as a critical factor in the development and progression of BC, with the canonical NF‐κB pathway playing a central role; it can also drive an immunosuppressive tumor microenvironment that compromises the efficacy of immunotherapy [15, 16, 17, 18]. *Akkermansia muciniphila* (AKK), a Gram‐negative bacterium first isolated from human feces in 2004, has demonstrated unique potential in regulating immune homeostasis and suppressing excessive inflammation [19, 20, 21]. It is reported that AKK can alleviate pathological inflammation, largely by suppressing NF‐κB‐related responses, with its outer membrane protein Amuc_1100 being a major mediator of these effects [22, 23, 24]. At the same time, AKK exhibits potent immune‐activating properties, promoting antigen cross‐presentation, effectively reversing the immunosuppressive tumor microenvironment (TME), and enhancing CD8^+^ T‐cell activation, thus driving anti‐tumor effects [25, 26]. Notably, heat‐inactivated AKK retains its strong immune‐stimulating activity, helping mitigate side effects associated with live bacterial therapies, making it a safer therapeutic option [20]. Recent advances in modified probiotics have further highlighted that physicochemical or biological modification can improve probiotic stability, retention, delivery capacity, and therapeutic efficacy, and can enable their integration with other therapeutic modalities for combinatorial treatment [27]. Therefore, combining the immunomodulatory effects of AKK as a bacterial formulation in BC treatment holds significant promise and potential.

**FIGURE 1:**
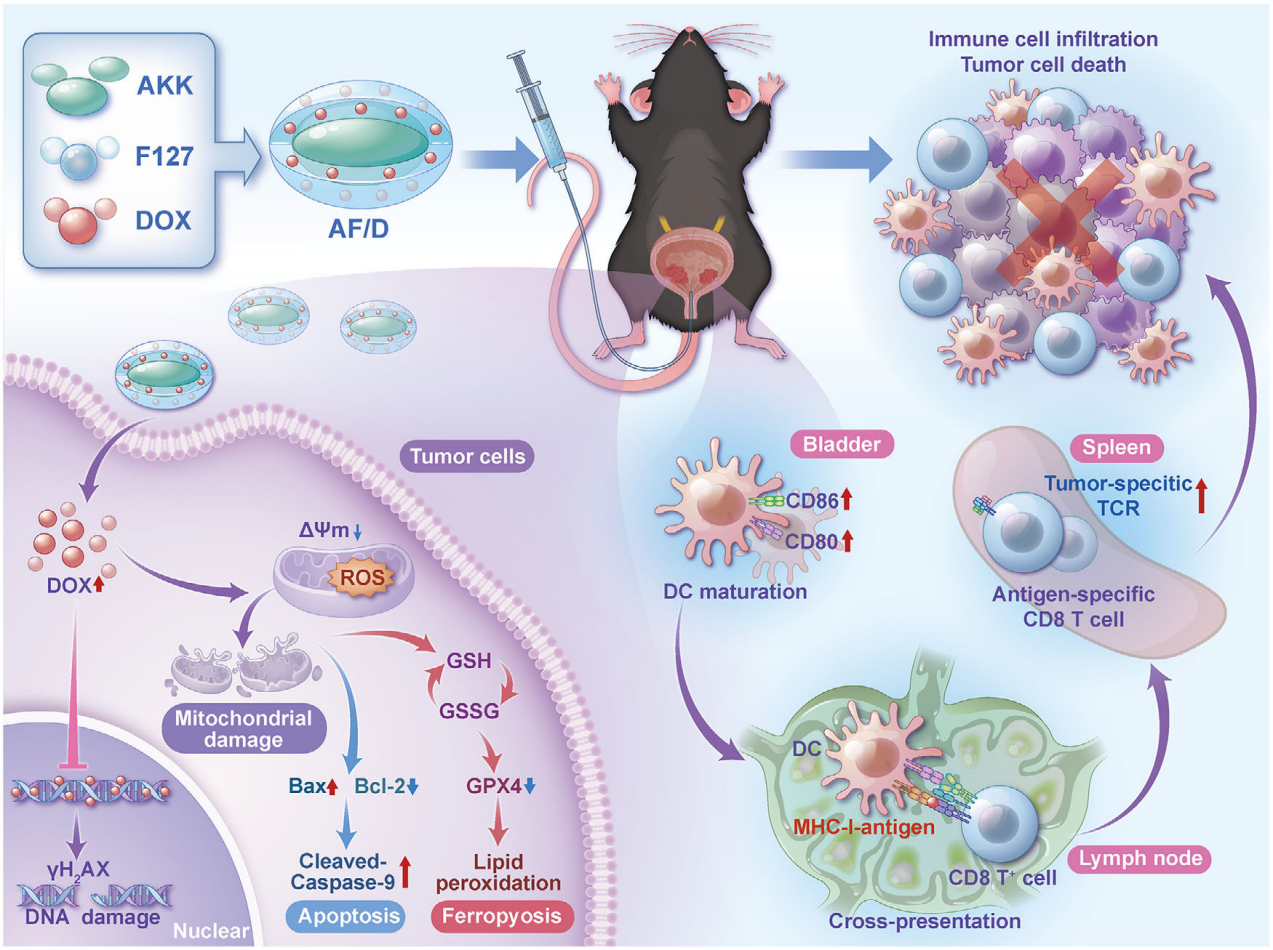
A novel intravesical, bacteria‐based delivery system (AF/D) that establishes a synergistic therapeutic strategy by integrating chemotherapy with probiotic‐based immunotherapy.

In this study, we developed a synergistic chemo‐immunotherapy for BC (Figure 1). Pasteurized AKK, a probiotic authorized as a novel food in the European Union [19], was conjugated with the FDA‐approved, biocompatible surfactant F127 [28]. Doxorubicin (DOX), a clinical chemotherapeutic agent for bladder cancer, was then efficiently loaded onto the AKK carrier via electrostatic adsorption, yielding the AKK‐F127/DOX (AF/D) formulation. Our results showed that AF/D exhibited significant anti‐tumor activity in vitro, activating stronger ferroptosis and apoptosis than free dox, characterized by calreticulin exposure, HMGB1 release, and ATP efflux. Moreover, AF/D induced immunogenic cell death (ICD) and downregulated the pro‐inflammatory NF‐κB pathway in tumor cells, promoted dendritic cells (DCs) maturation and M1‐like macrophage polarization, thereby enhancing antigen cross‐presentation and CD8^+^ T cell priming. In orthotopic models, intravesical AF/D surpassed all monotherapies and showed a significant improvement in survival. The treatment concurrently reshaped the TME by enhancing T cell infiltration and dendritic cell maturation, and generated a systemic antigen‐specific CD8^+^ T cell response in the spleen, while maintaining a favorable safety profile.

## 2 Results and Discussion

### 2.1 Preparation and Characterization of AF/D

**FIGURE 2:**
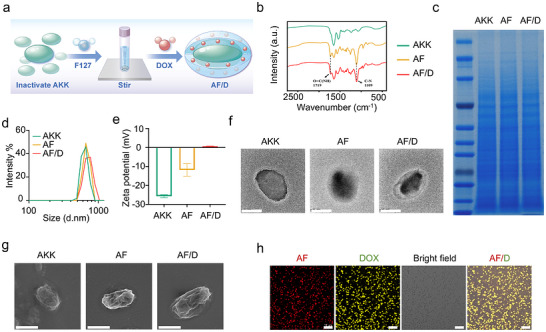
Preparation and characterization of AF/D. (a) Schematic overview of the fabrication of AF/D. (b) Fourier‐transform infrared spectroscopy (FT‐IR) spectra of AKK, AF, and AF/D. (c) SDS–PAGE profiles of proteins from AKK, AF, and AF/D. Samples were loaded at equal protein amounts and visualized by Coomassie blue staining. (d, e) Hydrodynamic size distribution (d) and zeta potential (e) of AKK, AF, and AF/D (n = 3). (f, g) Representative TEM (f) and SEM (g) images of AKK, AF, and AF/D. Scale bars, 500 nm. (h) Confocal laser scanning microscopy (CLSM) images confirming successful AF/D construction. Scale bars, 10 µm.

**FIGURE 3:**
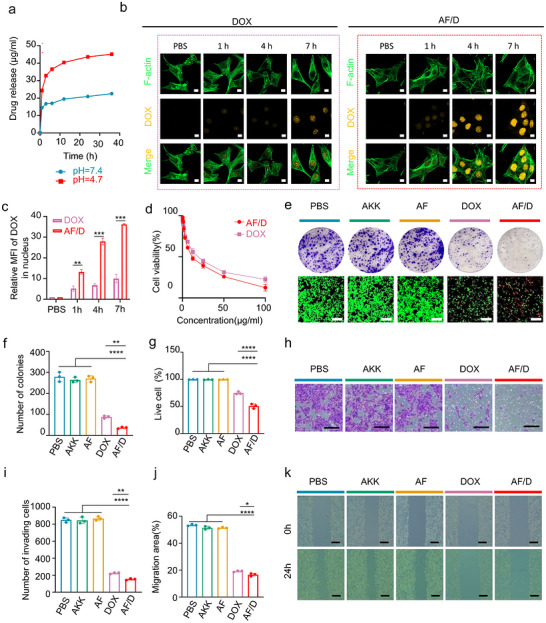
In vitro antitumor activity of AF/D. (a) pH‐dependent DOX release from AF/D under neutral pH and acidic urinary pH conditions, with pH 4.7 used to mimic acidic urine after intravesical administration. (b, c) CLSM images of MB49 cells incubated with free DOX or AF/D for 1, 4, and 7 h (b) and corresponding quantification of nuclear DOX fluorescence intensity (c). F‐actin was stained with phalloidin (green). Scale bars, 10 µm. (d) Cell viability of MB49 cells treated with varying concentrations of DOX or AF/D measured by CCK‐8 assay. (e–g) Colony formation of MB49 cells assessed by crystal violet staining (f, up) with semi‐quantitative analysis (e) and live/dead staining of MB49 cells (Calcein‐AM, green; PI, red) (f, down) and quantification of cell viability among different treatment groups (g). Scale bars, 40 µm. (h, i) Transwell invasion assay of MB49 cells following AF/D treatment. Representative images of MB49 cells that invaded to the underside of the Transwell membrane after exposure to AF/D or the indicated control treatments are shown(h), together with the corresponding quantitative analysis of invaded cell numbers (i). Scale bars, 100 µm. (j, k) In vitro wound‐healing (scratch) assay of MB49 cells. Representative optical micrographs and quantification of wound closure are shown. A linear scratch was introduced into confluent MB49 monolayers to generate a cell‐free gap (0 h). Cells were then incubated with AF/D or the indicated control treatments, and wound closure was assessed after 24 h. Scale bars, 100 µm.

The fabrication process of the AKK‐F127/DOX (AF/D) drug delivery platform is illustrated in Figure 2a. AKK was inactivated via pasteurization. The amine‐terminated F127 was conjugated with carboxyl groups on the AKK surface through EDCI‐mediated amide bond formation, yielding the AKK‐F127 (AF) complex. Subsequently, DOX was added and stirred to load the drug, yielding the bacteria‐based drug delivery system, AF/D. The successful synthesis and basic properties of the AF/D conjugate were systematically confirmed through a series of characterizations. First, FT‐IR spectroscopy verified the chemical conjugation between F127 and AKK, as evidenced by the appearance of new absorption peaks at 1109 m^−1^ (C─N) and 1719 cm^−1^ (amide bond) (Figure 2b). Importantly, this chemical modification did not compromise the protein integrity of AKK, as gel electrophoresis confirmed nearly identical protein profiles for AKK, AF, and AF/D (Figure 2c). In the physicochemical properties, the formation of AF/D resulted in a shift of zeta potential from −25.66 ± 0.70 mV (AKK) to a nearly neutral 0.65 ± 0.15 mV, accompanied by an increase in hydrodynamic diameter from 660 to 768 nm (Figure 2d,e), confirming the formation of the composite structure. To further evaluate the stability of AF/D, we monitored its hydrodynamic size distribution in buffer over time. AF/D maintained a similar size distribution profile at 0, 24, 48, and 72 h, with no obvious peak shift, broadening, or large aggregate formation (Figure S1). These results indicate that AF/D possesses good stability in buffer for at least 72 h. The morphological consequences of this assembly were directly visualized by electron microscopy. TEM images revealed that native AKK exhibited an intact, near‐spherical shape, while the AF complex showed a blurred periphery. Interestingly, the AF/D conjugate regained a distinct boundary, likely due to the high electron density of the loaded component (Figure 2f and Figure S2). These observations were supported by SEM, which confirmed the maintenance of overall bacterial structural integrity (Figure 2g). Having established the successful construction and altered morphology of the carrier, we then specifically verified the loading of DOX. UV–vis spectroscopy of AF/D showed a strong characteristic absorption peak at 480 nm, corresponding to free DOX, confirming the presence of the drug on the platform (Figure S3). The successful incorporation and spatial co‐localization of DOX within the carrier were further confirmed by confocal laser scanning microscopy (CLSM), as demonstrated by the overlapping fluorescence signals of DOX and the Cy5.5‐labeled AF complex (Figure 2h). Collectively, these results confirm the successful preparation of the AF/D conjugate. Furthermore, the conjugate exhibited a high drug loading efficiency of ∼85% (Figure S4). To clarify the advantage of F127 modification, we further prepared AKK/DOX without F127 modification, abbreviated as A/D, by directly mixing AKK with DOX through electrostatic adsorption. The DOX loading efficiency of A/D was approximately 33.77%, which was markedly lower than that of AF/D (∼85%) (Figure S4). This result indicates that F127 modification substantially improved the drug‐loading capacity of the AKK‐based platform. Also, AF/D exhibited a pH‐dependent release profile. The acidic condition of pH 4.7 was selected to mimic acidic urine after intravesical administration, as AF/D is designed to be directly exposed to urine in the bladder. The accelerated DOX release at pH 4.7 (Figure 3a) indicates an acid‐responsive release behavior under acidic urinary conditions. Since AF/D is locally administered into the bladder cavity, DOX released under acidic urinary conditions may still contribute to local chemotherapy at the tumor‐bearing bladder site rather than representing systemic drug loss.

### 2.2 In Vitro Cytotoxicity of AF/D

The therapeutic potential of the AF/D conjugate was first evaluated by assessing its cellular internalization and cytotoxicity against MB49 bladder cancer cells. Intracellular uptake analysis revealed a time‐dependent increase in DOX fluorescence following incubation with AF/D, confirming the efficient internalization of the nano‐bacterial platform (Figure 3b,c). After adding free DOX as a control, AF/D‐treated cells showed stronger intracellular and nuclear DOX fluorescence than free DOX‐treated cells at the corresponding time points, indicating that AF/D enhanced DOX uptake by MB49 bladder cancer cells. Flow cytometry further demonstrated that at DOX concentrations exceeding 1.6 µg/mL, the cellular uptake of AF/D was markedly higher than that of free DOX (Figure S5). This enhanced uptake may attribute to distinct entry mechanisms, where free DOX primarily relies on passive diffusion, while the larger AF/D particles may be internalized more efficiently via macropinocytosis or other high‐capacity endocytic pathways, which are often upregulated in cancer cells [29, 30, 31, 32, 33, 34, 35]. Notably, these uptake results demonstrate efficient internalization of AF/D by MB49 bladder cancer cells under the tested in vitro conditions, but do not indicate active tumor‐cell‐specific recognition.

The consequent antitumor effects were comprehensively evaluated. CCK‐8 assays showed that AKK alone did not significantly affect MB49 cell viability within the tested concentration range, whereas both free DOX and AF/D significantly reduced MB49 cell viability (Figure 3d and Figure S6), confirming effective drug delivery. Notably, AF/D exhibited stronger cytotoxicity than free DOX at equivalent doses. This enhanced potency was corroborated by colony formation assays, where AF/D more potently inhibited long‐term clonogenic survival than free DOX (Figure 3e,f). Live/dead staining (Calcein‐AM/PI) provided direct visual evidence that AF/D treatment resulted in a higher proportion of PI‐positive (dead) cells compared to free DOX treatment, aligning with the viability data (Figure 3e,g).

Beyond direct cell killing, AF/D significantly impaired the aggressive behaviors of MB49 cells. In a wound‐healing assay, AF/D treatment led to the smallest wound closure rate, indicating a strong inhibition of cell migration (Figure 3h,i). Furthermore, in a Matrigel‐based transwell invasion assay, AF/D treatment resulted in the lowest number of invasive cells, demonstrating a potent suppression of metastatic potential (Figure 3j,k). Taken together, these results demonstrate that the AF/D conjugate exerting stronger cytotoxic, anti‐proliferative, and anti‐metastatic effects than free DOX alone.

### 2.3 Apoptosis, Ferroptosis Enabled by AF/D

**FIGURE 4:**
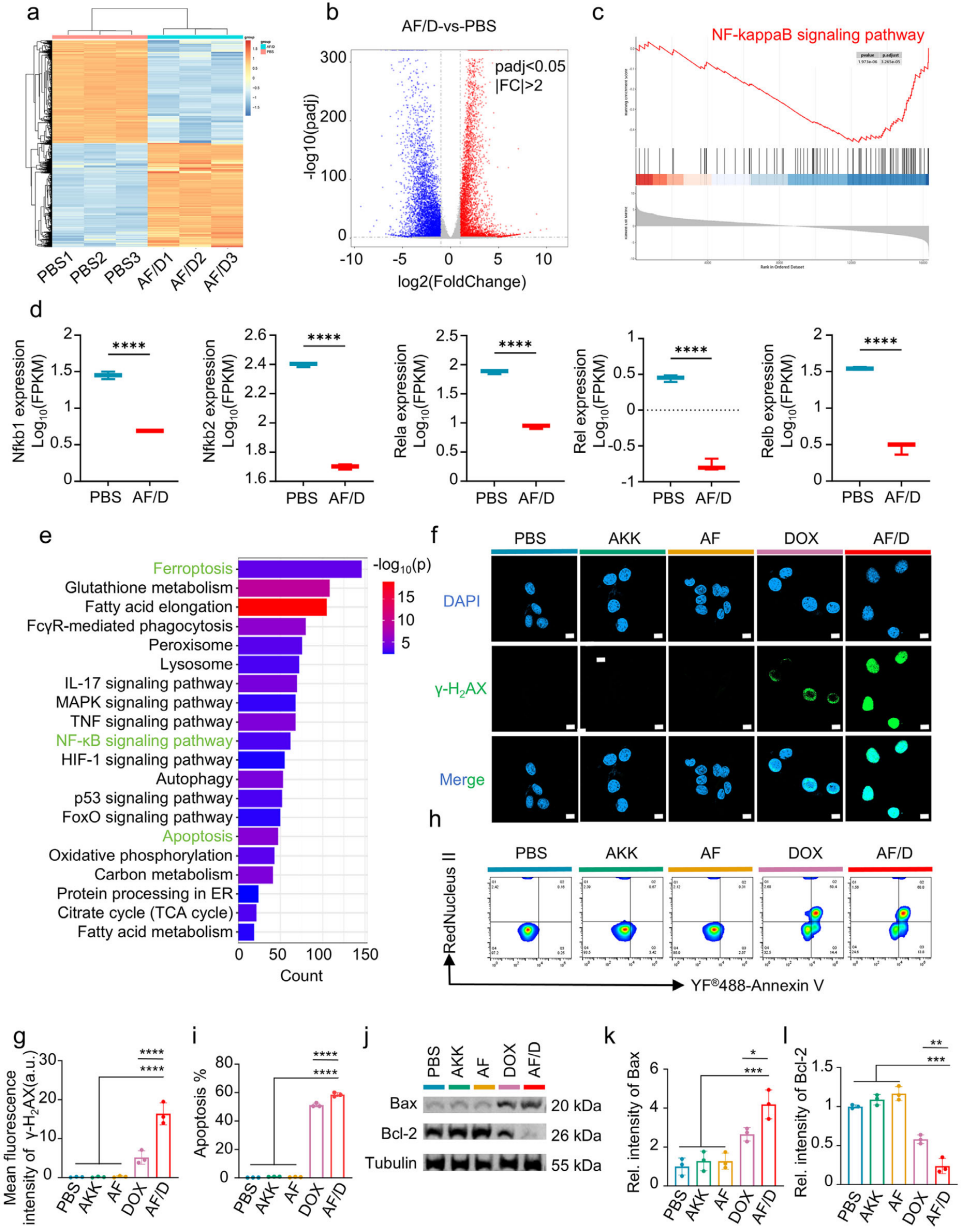
AF/D treatment downregulates NF‐κB signaling and induces apoptosis in MB49 bladder cancer cells. (a) Clustered heatmap showing differential gene expression patterns between PBS‐ and AF/D‐treated MB49 cells. (b) Volcano plot illustrating differentially expressed genes between the two groups. Significantly upregulated and downregulated genes were defined using the criteria of |FoldChange| > 2 and adjusted p value (padj) < 0.05. (c) GSEA plot indicating that the NF‐κB signaling pathway is significantly downregulated in AF/D compared with PBS (*p* = 1.973 × 10^−6^; adjusted *p* = 3.265 × 10^−5^). (d) Downregulation of key NF‐κB pathway genes in MB49 cells after AF/D treatment. RNA‐seq analysis was used to compare the expression of representative NF‐κB signaling components (Nfkb1, Nfkb2, Rela, Rel, and Relb) between the PBS and AF/D groups. Gene expression is presented as log10 (FPKM). (e) KEGG pathway enrichment of differentially expressed genes in MB49 cells treated with PBS vs. AF/D. (f, g) CLSM images of γ‐H_2_AX in MB49 cells following different treatments (f) and corresponding fluorescence quantification (g). Scale bars, 100 µm. (h, i) FCM plots (h) and quantification (i) of apoptosis in MB49 cells across treatment groups. (j) Western blot analysis of Bax and Bcl‐2 expression in MB49 cells, with tubulin as the loading control. (k, l) Densitometric quantification of Bax (k) and Bcl‐2 (l) levels based on grayscale analysis.

To explore the molecular alterations induced by AF/D treatment, we performed transcriptome analysis of MB49 cells treated with AF/D vs. PBS. This comparison was designed to identify the major AF/D‐associated transcriptional programs rather than to distinguish the individual transcriptomic contributions of DOX and AKK. A total of 9610 differentially expressed genes (DEGs) were identified between the two groups using the criteria of |FoldChange| > 2 and adjusted *p*‐value (padj) < 0.05, as illustrated in the clustered heatmap (Figure 4a). The full list of DEGs is provided in Table S1. The volcano plot further details these changes, showing 4832 upregulated and 4778 downregulated genes in the AF/D‐treated group (Figure 4b). Therefore, the transcriptomic data should be interpreted as an exploratory analysis of AF/D‐induced molecular changes relative to PBS. The advantages of AF/D over free DOX or AKK alone were further evaluated through functional assays, including cytotoxicity, apoptosis/ferroptosis induction, ICD, antigen cross‐presentation, and in vivo therapeutic efficacy.

Given the pivotal role of chronic inflammation in bladder carcinogenesis [15, 16, 17, 18], a process in which the NF‐κB pathway is closely associated with the initiation and progression of bladder cancer [36, 37], we further examined the effect of AF/D on NF‐κB signaling. Gene Set Enrichment Analysis (GSEA) showed that the NF‐κB signaling pathway was significantly downregulated in AF/D‐treated MB49 cells compared with PBS‐treated cells (Figure 4c). Consistently, key molecules within the NF‐κB pathway, including Nfkb1, Nfkb2, Rela, Relb, and Rel, were significantly downregulated in the AF/D group compared with the PBS control (Figure 4d). This observed suppression aligns with the known anti‐inflammatory properties of AKK [38, 39], suggesting that inhibition of the NF‐κB signaling pathway may be one of the mechanisms through which AF/D exerts its antitumor effects.

KEGG pathway enrichment analysis of these DEGs revealed significant enrichment of apoptosis and ferroptosis pathways, along with other pathways involved in redox and metabolic regulation, including glutathione metabolism, NF‐κB signaling, and MAPK signaling (Figure 4e). GO analysis of the differentially expressed genes is shown in Figure S7. Ferroptosis, a form of regulated cell death driven by lipid peroxidation, is closely linked to these redox‐ and metabolism‐related pathways such as glutathione metabolism, NF‐κB, and MAPK signaling [40, 41, 42, 43, 44, 45, 46, 47, 48, 49, 50]. Given the central roles of these two death pathways in tumor progression, we focused subsequent experimental validation on apoptosis and ferroptosis to determine their potential mediation of AF/D's antitumor effects.

Building on the transcriptomic findings that implicated both apoptosis and ferroptosis, we first investigated the pro‐apoptotic mechanism of AF/D. DOX is known to induce DNA damage, a key trigger of apoptosis [51, 52]. Immunostaining for γ‐H_2_AX confirmed that AF/D treatment indeed caused significant DNA damage in MB49 cells (Figure 4f,g). This damage could initiate mitochondrial stress and ROS elevation, leading to apoptosis [53, 54, 55, 56]. Consistent with this, flow cytometry revealed that AF/D induced a higher apoptotic rate (60.0%) than free DOX (51.6%), while AKK alone was ineffective (Figure 4h,i).

To delineate the apoptotic pathway, we investigated the mitochondria‐mediated intrinsic pathway, a key mechanism of DOX‐induced cell death [57, 58]. Western blot analysis of key proteins in this pathway revealed that AF/D most potently upregulated the pro‐apoptotic protein Bax and downregulated the anti‐apoptotic Bcl‐2 compared to all other groups (Figure 4j–l and Figure S8). This shift in the Bax/Bcl‐2 ratio toward a pro‐apoptotic state indicates the activation of the mitochondrial pathway.

**FIGURE 5:**
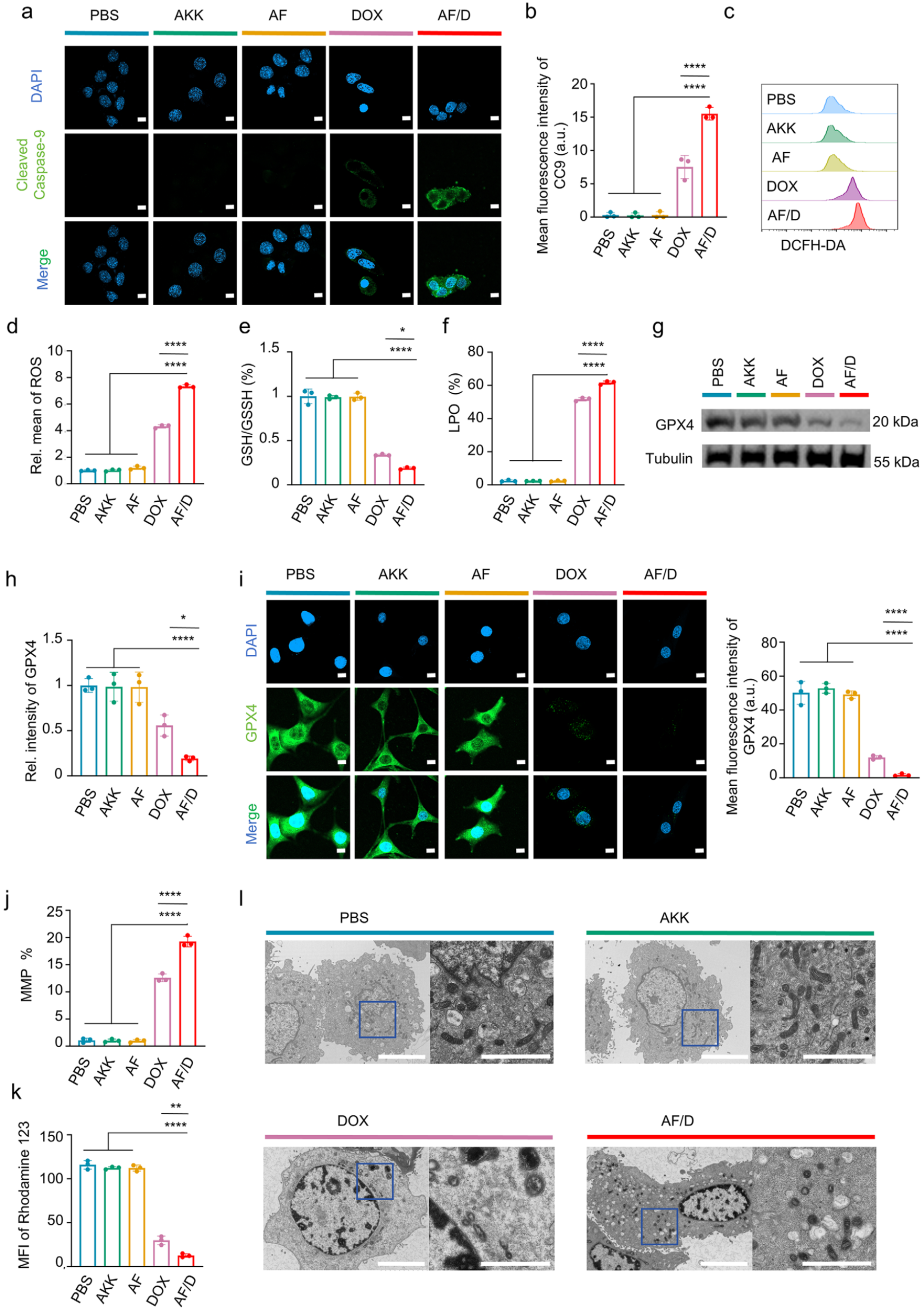
AF/D treatment induces ferroptosis and mitochondrial damage in MB49 bladder cancer cells. (a, b) CLSM images of cleaved caspase‐9 (CC9) in MB49 cells following different treatments (a) and corresponding fluorescence quantification (b). Scale bars, 100 µm. (c) FCM profiles demonstrating intracellular ROS levels in MB49 cells under different treatments. (d) Intracellular ROS levels measured by FCM in MB49 cells after the indicated treatments. (e) Relative GSH/GSSG levels in MB49 cells subjected to different treatments. (f) Lipid peroxidation in MB49 cells assessed by FCM following various treatments. (g, h) Western blot analysis of GPX4 expression in MB49 cells, with tubulin as the loading control (g). Densitometric analysis of GPX4 expression (h). (i) CLSM images of GPX4 in MB49 cells following different treatments and corresponding fluorescence quantification. Scale bars, 100 µm. (j) FCM analysis and quantification of mitochondrial membrane potential (MMP) changes in MB49 cells after different treatments using Rhodamine 123 staining. (k) Quantification of Rhodamine 123 fluorescence intensity corresponding to the CLSM images after different treatments. Rhodamine 123 accumulates in mitochondria in a membrane potential‐dependent manner; therefore, reduced Rhodamine 123 fluorescence indicates mitochondrial membrane potential loss and mitochondrial dysfunction. (l) TEM images of MB49 cells treated with PBS, AKK, DOX or AF/D, showing pronounced mitochondrial damage in the AF/D group. Scale bars, 5 µm (main images) and 2 µm (magnified views).

Mitochondrial apoptotic pathway activation is known to trigger downstream Caspase‐9 cleavage [59]. Consistently, AF/D most potently increased cleaved Caspase‐9 levels as shown by western blot analysis (Figure S9), and the enhanced activation and distinct localization of cleaved Caspase‐9 in AF/D‐treated cells were further corroborated by confocal microscopy (Figure 5a,b). Taken together, these data demonstrate that AF/D induces DNA damage and activates the mitochondrial apoptotic pathway more effectively than free DOX.

Building on the observation that AF/D induces DNA damage and mitochondrial stress, both of which are key events driving reactive oxygen species (ROS) overproduction, we further explored its role in triggering ferroptosis, as initially suggested by transcriptomic analysis. As a key upstream event in this cascade, the overproduction of ROS was verified, with AF/D triggering a more pronounced increase in intracellular levels than free DOX (Figure 5c,d). Concomitantly, a more severe depletion of glutathione (GSH) was observed in the AF/D‐treated group (Figure 5e). Since lipid peroxidation (LPO) is the hallmark execution event of ferroptosis [60], we measured LPO and found that AF/D induced the highest level among all treatments (Figure 5f and Figure S10). Meanwhile, the expression of glutathione peroxidase 4 (GPX4), a key negative regulator of ferroptosis [61], was downregulated more significantly by AF/D than by free DOX, as shown by western blotting and immunofluorescence (Figure 5g–i).

Mitochondrial damage serves as a critical node linking both apoptotic and ferroptotic pathways. Assessment of mitochondrial membrane potential (ΔΨm) using Rhodamine 123 staining revealed that AF/D induced a much more severe depolarization than free DOX (Figure 5j,k and Figures S11 and S12). TEM showed marked pathological changes in the mitochondria of AF/D‐treated cells, including cristae loss and swelling (Figure 5l). These pathological alterations in mitochondrial ultrastructure corroborated the loss of ΔΨm and highlighted the central role of mitochondrial damage in the execution of cell death.

NF‐κB inhibition may be associated with AF/D‐induced apoptosis and ferroptosis. As a pro‐survival pathway, NF‐κB can promote the expression of anti‐apoptotic proteins and thereby protect tumor cells from stress‐induced mitochondrial apoptosis [37, 62, 63]. Thus, AF/D‐mediated NF‐κB suppression may weaken anti‐apoptotic signaling, consistent with Bax upregulation, Bcl‐2 downregulation, and cleaved caspase‐9 activation. NF‐κB signaling also participates in oxidative stress regulation and therapy resistance, which may affect ferroptosis sensitivity [37, 64, 65]. Therefore, the observed NF‐κB inhibition may contribute to AF/D‐induced ROS accumulation, GSH depletion, lipid peroxidation, and GPX4 downregulation. However, the current data support a potential association rather than a direct causal relationship between NF‐κB inhibition and AF/D‐induced apoptosis/ferroptosis.

The stronger ferroptosis induction by AF/D compared with free DOX is unlikely to be explained solely by higher intracellular DOX accumulation. Although enhanced uptake of AF/D increases intracellular DOX exposure and may promote ROS generation, AF/D also induced a coordinated disruption of ferroptosis‐related defense mechanisms, including more severe GSH depletion, enhanced lipid peroxidation, and stronger GPX4 downregulation. These changes indicate that AF/D weakens the GSH/GPX4 antioxidant axis and thereby sensitizes MB49 cells to lipid peroxidation‐driven ferroptosis. In addition, AF/D caused more pronounced mitochondrial membrane potential loss and mitochondrial structural damage, which may further amplify oxidative stress. The observed suppression of NF‐κB signaling may also reduce pro‐survival and anti‐stress responses, contributing to increased ferroptosis susceptibility. Therefore, AF/D‐induced ferroptosis may result from the combined effects of enhanced DOX delivery, redox imbalance, GPX4 inhibition, mitochondrial dysfunction, and NF‐κB pathway suppression.

In summary, AF/D induces potent mitochondrial damage, which concurrently amplifies the intrinsic apoptotic pathway and triggers ferroptosis. This dual activation of distinct cell death modalities underlies the potential antitumor efficacy of the AF/D conjugate.

### 2.4 AF/D Induces Immunogenic Cell Death and Elicits Antigen‐Specific T Cell Responses

**FIGURE 6:**
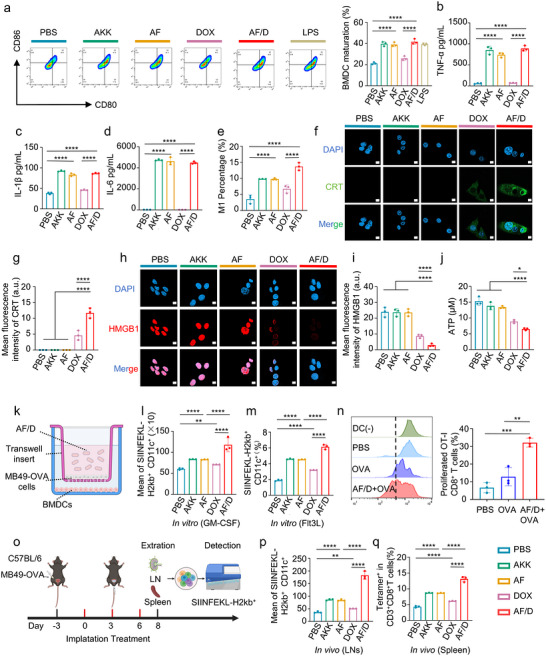
AF/D promotes immune activation and enhances antigen cross‐presentation. (a) FCM analysis of BMDC maturation following different treatments. LPS represents lipopolysaccharide, which was used as a positive control for immune stimulation. (b–d) Concentrations of pro‐inflammatory cytokines, including tumor necrosis factor‐α (TNF‐α) (b), interleukin‐1β (IL‐1β) (c), and interleukin‐6 (IL‐6) (d), in BMDC culture supernatants. (e) FCM analysis of M1 macrophage polarization in RAW264.7 cells under different treatments. (f, g) CLSM images showing calreticulin (CRT) surface exposure on MB49 cells after the indicated treatments (f), with corresponding fluorescence quantification (g). Scale bars, 10 µm. (h, i) CLSM images of HMGB1 release in treated MB49 cells (h) and associated quantitative analysis (i). Scale bars, 10 µm. (j) Intracellular ATP levels in MB49 cells following different treatments. (k) Schematic diagram of the Transwell‐based MB49‐OVA/BMDC co‐culture system used to assess antigen transfer and cross‐presentation in vitro. (l, m) Quantification by FCM of SIINFEKL–H‐2K^b^ presentation on BMDCs differentiated with GM‐CSF (l) or Flt3L (m) followed by different treatments. (n) Representative FCM plots and statistical analysis of OT‐I CD3^+^CD8^+^ T‐cell proliferation assessed by CFSE dilution after co‐culture with BMDCs pulsed with PBS, OVA, or AF/D + OVA. (o) Schematic overview of the in vivo dosing schedule for antigen cross‐presentation studies. (p) FCM analysis of SIINFEKL–H‐2K^b^ in DCs isolated from draining lymph nodes. (q) Statistical data of the H‐2Kb/SIINFEKL tetramer staining of CD3^+^CD8^+^ T cells in the spleen.

To objectively distinguish the individual contributions of AKK‐mediated immunostimulation and DOX‐induced immunogenic cell death (ICD), we first evaluated the direct effects of the different formulations on bone marrow‐derived dendritic cells (BMDCs). AKK, AF, and AF/D effectively promoted BMDC maturation, whereas free DOX showed little direct stimulatory activity under the same experimental conditions (Figure 6a). Consistently, AKK, AF, and AF/D stimulated BMDCs to secrete TNF‐α, IL‐1β, and IL‐6, whereas free DOX induced limited cytokine secretion (Figure 6b–d). These results indicate that the AKK component provides direct bacteria‐derived adjuvant‐like signals capable of activating BMDCs. Furthermore, AF/D promoted the polarization of RAW264.7 macrophages toward the M1‐like phenotype (Figure 6e and Figure S13).

Because DOX‐induced ICD may activate dendritic cells indirectly through tumor‐cell‐derived DAMPs and other soluble immunogenic mediators, we next performed a tumor‐cell‐conditioned‐medium experiment. MB49 cells were treated with PBS, AKK, AF, DOX, or AF/D for 24 h, after which the culture supernatants were collected and transferred to BMDC cultures. Flow cytometric analysis showed that conditioned medium from DOX‐treated MB49 cells increased the proportion of mature BMDCs compared with conditioned medium from PBS‐treated cells (Figure S14). This finding indicates that DOX can contribute to dendritic‐cell activation indirectly through soluble immunogenic signals released from treated tumor cells. Conditioned media from AKK‐ and AF‐treated MB49 cells also promoted BMDC maturation, which may reflect contributions from soluble AKK‐derived immunostimulatory components and tumor‐cell‐derived signals. Among the tested groups, conditioned medium from AF/D‐treated MB49 cells induced the highest level of BMDC maturation. Together, these results demonstrate that DOX‐induced ICD and AKK‐mediated immunostimulation contribute to immune activation through distinct but complementary mechanisms. DOX primarily provides tumor‐cell‐derived DAMPs and antigenic material through ICD, whereas AKK provides direct bacteria‐derived adjuvant‐like stimulation. Thus, the coexistence of ICD and AKK is not strictly required for immune activation. Instead, their integration within the AF/D platform may coordinate tumor antigen release with dendritic‐cell activation, thereby enhancing antigen cross‐presentation and downstream CD8^+^ T‐cell priming.

Having established that AF/D directly elicits innate immune activation, we next examined its capacity to generate the critical signals linking innate immune sensing to adaptive anti‐tumor immunity. Specifically, we assessed whether AF/D could induce immunogenic cell death (ICD), a process that liberates tumor antigens and robust danger signals to initiate immune surveillance. In MB49 bladder cancer cells, AF/D treatment could induce hallmarks of ICD, including robust cell surface exposure of calreticulin (an “eat‐me” signal), pronounced translocation and extracellular release of high‐mobility group box 1 (HMGB1, a key damage‐associated molecular pattern), and significant efflux of ATP (a critical “find‐me” signal). All these effects were markedly enhanced compared to free DOX treatment (Figure 6f–j). These results demonstrate that AF/D, through the synergy of apoptosis and ferroptosis, efficiently converts tumor cell death into an immunogenic event, furnishing tumor antigens and DAMPs for immune recognition. Although free DOX also induced several ICD‐associated signals, DOX alone did not directly activate BMDCs in our direct stimulation assay. Although free DOX showed limited direct stimulation of BMDCs, conditioned medium from DOX‐treated MB49 cells promoted BMDC maturation, indicating that DOX contributes to immune activation indirectly through ICD‐associated tumor‐cell‐derived signals. In contrast, AKK‐based formulations provide direct bacteria‐derived immunostimulatory signals. AF/D integrates these complementary routes by coupling tumor antigen and DAMP release with direct dendritic‐cell stimulation.

Potent antitumor immune‐mediated cytotoxicity relies on effective antigen presentation and CD8 T‐cell activation. Therefore, with validation that AF/D both activates antigen‐presenting cells and generates immunogenic tumor material, we next sought to determine whether these two processes are functionally coupled in driving tumor‐specific CD8 T cell priming. To model this functional coupling, we established a Transwell‐based co‐culture system. In this setup, MB49‐OVA tumor cells (upper chamber) were treated with AF/D or controls, while bone marrow‐derived dendritic cells (BMDCs) were cultured in the lower chamber, simulating in vivo antigen transfer (Figure 6k). AF/D treatment led to a significant increase in the OVA cross‐presentation capacity of BMDCs compared to PBS or free DOX, a result consistent across BMDCs generated with either GM‐CSF or Flt3L, which induce distinct DC subsets (Figure 6l,m). This demonstrates that AF/D‐generated immunogenic signals from tumor cells are efficiently processed and presented by DCs. Critically, this enhanced cross‐presentation translated into functional T‐cell activation. In an OT‐I CD8^+^ T‐cell priming assay, BMDCs that had been co‐cultured with AF/D‐treated tumor cells induced markedly stronger proliferation of antigen‐specific CD8^+^ T cells compared to controls (Figure 6n). These data conclusively show that the ICD induced by AF/D and the DC activation it triggers are functionally integrated to prime a strong, tumor‐antigen‐specific cytotoxic T‐cell response in vitro.

To evaluate whether the immunomodulatory effects of AF/D observed in vitro may translate to a therapeutic context, we assessed its efficacy in an MB49‐OVA bladder tumor mouse model. Mice received intravesical instillations of different treatments according to the regimen outlined in Figure 6o. Analysis of immune cells from treated mice revealed that AF/D treatment led to the most efficient cross‐presentation of the OVA on dendritic cells within the tumor‐draining lymph nodes (Figure 6p), demonstrating that the platform's ability to enhance this key process is functional in vivo. This enhanced cross‐presentation was further corroborated by a corresponding increase in the frequency of SIINFEKL‐MHC I tetramer‐positive CD8^+^ T cells in the spleens of the same mice (Figure 6q), confirming the successful generation of tumor antigen‐specific cytotoxic T lymphocytes. Together, these complementary events underscore that AF/D effectively coordinates local and systemic anti‐tumor immunity.

### 2.5 Intravesical Retention, Urinary Clearance, and Tumor Uptake of AF/D

To further investigate the local pharmacokinetic behavior of AF/D after intravesical administration, we evaluated its urinary clearance, bladder retention, and tumor tissue uptake. AF/D containing DOX at 1 mg/mL was intravesically administered at a volume of 50 µL, followed by urethral clamping for 2 h. The time point immediately after releasing the clamp was defined as 0 h. Urinary DOX concentrations were quantified by HPLC. The urinary DOX concentration was highest at 0 h and then rapidly decreased during the early phase, from approximately 102 914 ng/mL at 0 h to 10 888 ng/mL at 1 h and 72.7 ng/mL at 3 h (Figure S15). Low levels of DOX remained detectable up to 48 h after administration, suggesting early urinary clearance accompanied by sustained low‐level local DOX release from AF/D. Because urinary drug concentration can be influenced by urine volume and sampling variability, these data were interpreted together with bladder retention imaging.

In vivo fluorescence imaging of Cy5.5‐labeled AF/D showed that AF/D fluorescence remained detectable in the bladder region for up to 48 h, indicating prolonged intravesical retention (Figure S16). To further clarify the contribution of F127 modification, we compared the intravesical retention of Cy5.5‐labeled A/D and AF/D after bladder perfusion. A/D, prepared by direct electrostatic adsorption of DOX onto AKK without F127 modification, showed detectable fluorescence in the bladder region at early time points; however, the signal gradually weakened after 8–12 h and became faint at 24–48 h (Figure S16). In contrast, AF/D maintained a stronger and more persistent bladder fluorescence signal, with detectable fluorescence remaining even at 48 h. Together with the higher DOX loading efficiency of AF/D compared with A/D, these findings demonstrate that F127 modification improves both drug‐loading capacity and intravesical retention, thereby supporting its incorporation into the AF/D platform.

Furthermore, bladder tissue fluorescence imaging showed that DOX fluorescence was detectable in bladder tumor tissues from 0 h and remained visible at 0.5, 1, 2, 4, and 8 h after release of urethral clamping (Figure S17). These results demonstrate that AF/D can remain in the bladder after perfusion and that AF/D‐derived DOX can rapidly reach bladder tumor tissues during the intravesical retention period.

### 2.6 Intravesical AF/D Suppresses Orthotopic Bladder Tumor Growth In Vivo

**FIGURE 7:**
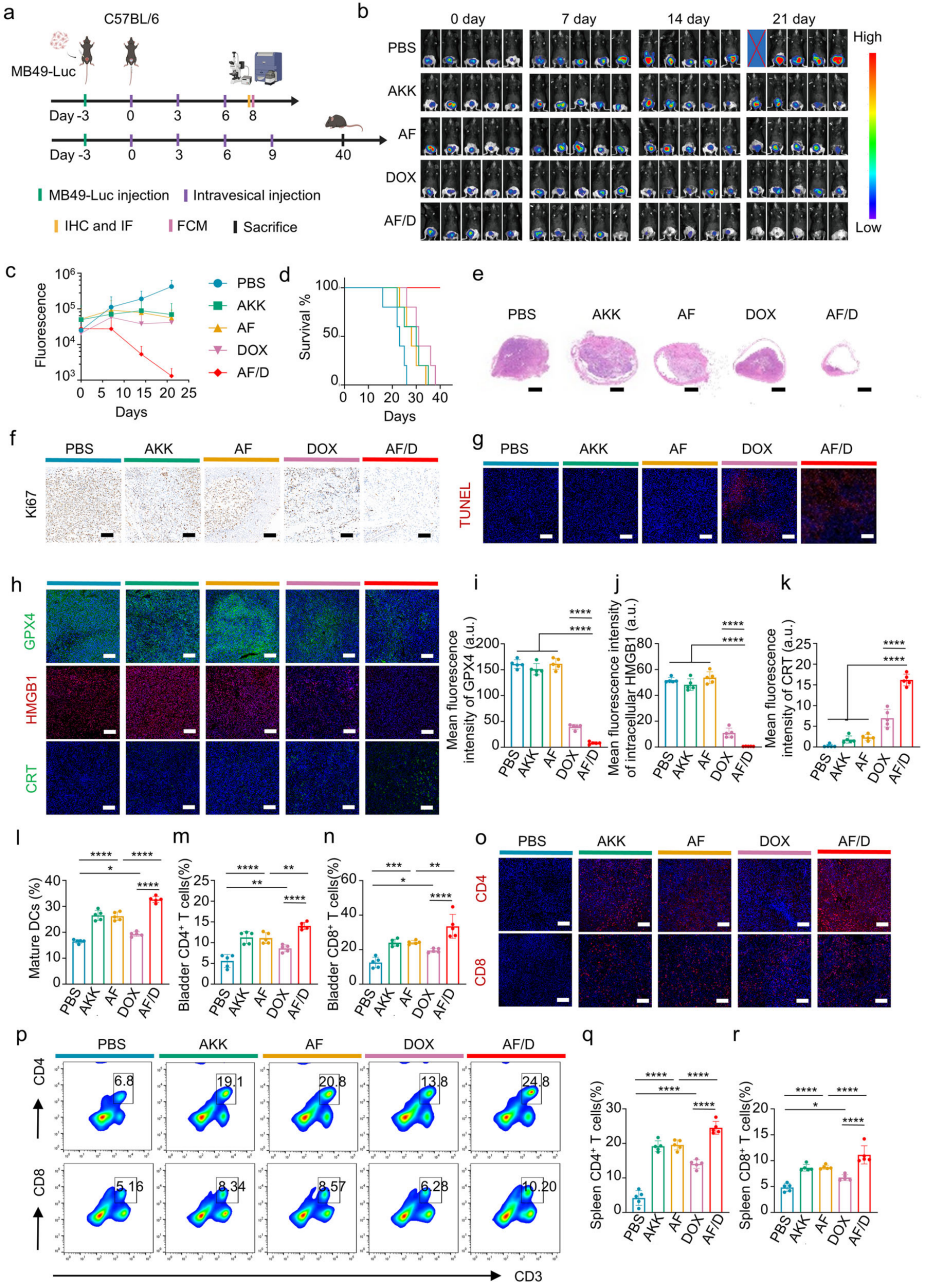
AF/D suppresses tumor growth and activates antitumor immunity in vivo. (a) Time line of the experiment in the orthotopic bladder cancer model. (b) Bioluminescence images of tumor‐bearing mice during intravesical administration of PBS, AKK, AF, DOX, or AF/D. Within each treatment group, mice are presented in the same left‐to‐right order across all time points. The red cross indicates that the corresponding mouse was died at that time point, and its original position was retained to preserve the longitudinal order. (c) Quantitative analysis of bladder tumor bioluminescence intensity based on region‐of‐interest signals from each mouse, presented as mean ± SD for each group. (e) Representative H&E‐stained sections of bladder tumors collected from mice receiving different treatments. Scale bars, 100 µm. (f, g) Ki‐67 immunostaining (f) and TUNEL staining (g) of tumor tissues from the indicated groups. Scale bars, 100 µm. (h) Representative immunofluorescence images showing GPX4 expression, intracellular HMGB1 retention, and CRT exposure in bladder tumor sections after the indicated treatments. Scale bars, 100 µm. (i–k) Quantitative analysis of GPX4 (i), HMGB1(j), and CRT (k) fluorescence intensities in tumor tissues from each group. (l) Flow cytometry–based quantification of DC maturation in bladder tumors across treatment groups. (m, n) Flow cytometry–based quantification of CD4^+^ (m) and CD8^+^ (n) T‐cell infiltration in the bladder across treatment groups. (o) Immunofluorescence staining of CD4 and CD8 in tumor sections from mice treated with the different formulations. Scale bars, 100 µm. (p–r) Representative flow cytometry plots(p), quantification of CD4^+^ T cells (q) and CD8^+^ T cells (r) in the spleens.

To evaluate the in vivo antitumor efficacy of AF/D, a formulation that integrates direct tumor cytotoxicity and bacteria‐driven immune activation, we established an orthotopic bladder tumor model in C57BL/6 mice using MB49‐LUC cells. Tumor‑bearing mice were randomized into five groups (PBS, AKK, AF, DOX, and AF/D) and treated by intravesical instillation following the protocol shown in Figure 7a. AF/D exhibited superior synergistic antitumor efficacy compared to all monotherapy groups. In vivo bioluminescence imaging demonstrated that AF/D most robustly suppressed tumor growth, as evidenced by the marked reduction in bladder tumor bioluminescence signals. In contrast, AKK, AF, or DOX monotherapy produced only partial tumor suppression, with fluorescence signals remaining detectable during the observation period (Figure 7b,c). Quantitative analysis of the region‐of‐interest bioluminescence intensity from each mouse confirmed that AF/D achieved the strongest inhibition of tumor burden among all treatment groups. Consistently, AF/D treatment significantly prolonged survival and achieved the highest survival rate (Figure 7d). Histological analysis further corroborated this marked synergy, with AF/D treatment eliciting the most profound tumor regression (H&E staining), the strongest suppression of proliferation (Ki67 staining), and the most extensive apoptotic cell death (TUNEL staining) (Figure 7e–g).

### 2.7 AF/D Remodels the Tumor Microenvironment and Elicits Systemic Antitumor Immunity In Vivo

The enhanced antitumor efficacy of AF/D correlated with the most pronounced induction of markers associated with both ferroptosis and ICD in vivo. These were not fully activated by any monotherapy. Immunofluorescence analysis showed that, compared with PBS, AKK, AF, or DOX treatment alone, AF/D most significantly downregulated GPX4 expression in tumor tissues (Figure 7h,i), and promoted HMGB1 release, as indicated by markedly reduced intracellular/nuclear HMGB1 retention in tumor sections (Figure 7h,j). Since HMGB1 is normally retained in the nucleus but is released extracellularly during ICD, the decreased intracellular HMGB1 fluorescence in the AF/D group supports the strongest induction of ICD by AF/D. Also, AF/D most significantly enhanced CRT exposure (Figure 7h,k). These results indicate that AF/D synergistically activates ferroptosis and ICD in vivo, a mechanism that extends beyond DOX‐induced apoptosis and underlies its enhanced efficacy.

To comprehensively assess the therapeutic effects of AF/D in vivo, we next examined its ability to modulate the TME and systemic immunity. Consistent with our in vitro findings, AF/D exhibited potent immunomodulatory functions in vivo. Flow cytometric analysis revealed that, among all treatment groups (PBS, AKK, AF, DOX), AF/D most effectively activated innate immune sentinel cells. It induced the highest proportion of mature DCs (Figure 7l and Figure S18) and most effectively drove macrophage polarization toward the M1 phenotype (Figures S19 and S20) in tumors, thereby reversing the local immunosuppressive milieu. This robust activation of the innate immune response subsequently led to a broad and potent adaptive immune response. Within the local bladder TME, AF/D treatment significantly enhanced the infiltration of both CD4^+^ and CD8^+^ T cells (Figure 7m–o and Figure S21), underscoring the advantage of intravesical instillation in eliciting potent on‐site anti‐tumor immunity. Systemic analysis further corroborated these findings, demonstrating that the proportions of activated CD4^+^ and CD8^+^ T cells in the spleens of the AF/D group were significantly higher than in all other treatment groups (Figure 7p–r). Notably, while AKK and AF monotherapies showed significant immunostimulatory activity, their combination with DOX in the AF/D formulation produced a clear synergistic effect, further amplifying the overall antitumor response. In summary, AF/D synergistically enhanced antigen cross‐presentation, potently activated innate immune cells, and significantly expanded antigen‐specific T cells both locally within tumors and systemically. These results collectively demonstrate that AF/D initiates a coherent immunostimulatory cascade, thereby establishing an effective systemic anti‐tumor immune response, which provides crucial mechanistic support for its superior therapeutic efficacy. Although AF/D enhanced antigen cross‐presentation and increased antigen‐specific T‐cell responses, the present study primarily demonstrates that AF/D activates systemic antitumor immunity and may provide an immunological basis for potential immune memory formation, but does not directly evaluate durable immunological memory or recurrence prevention.

Compared with simple co‐administration of free DOX and AKK, AF/D provides a co‐delivery formulation that spatially associates DOX with the F127‐modified AKK platform. This design may help maintain synchronized local exposure of the chemotherapeutic and immunomodulatory components in the bladder. In addition, AF/D showed higher DOX loading efficiency and prolonged intravesical retention, with detectable bladder fluorescence up to 48 h after instillation. DOX fluorescence was also detectable in bladder tumor tissues after perfusion, supporting local tumor exposure of AF/D‐derived DOX. These formulation‐related advantages may contribute to the stronger tumor cell killing, ICD induction, antigen cross‐presentation, CD8^+^ T‐cell priming, and in vivo antitumor efficacy observed with AF/D.

Collectively, these results demonstrate that the AF/D‐based intravesical instillation system can effectively remodel the bladder TME, elicits favorable local and systemic immune reprogramming, and thereby achieves great therapeutic efficacy against BC.

### 2.8 Biosafety Evaluation of AF/D In Vivo

**FIGURE 8:**
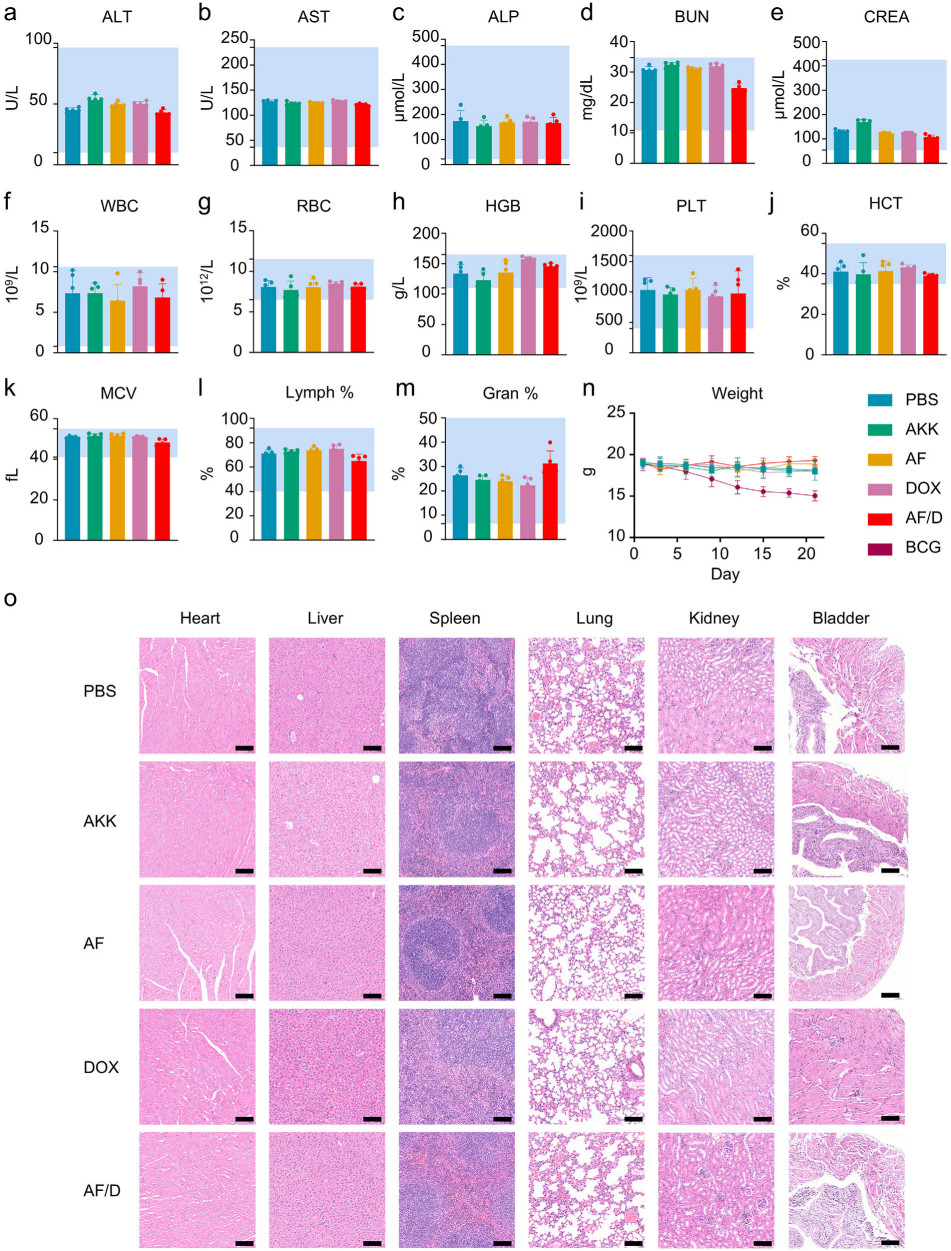
Biosafety evaluation of intravesical treatments. (a‐m) Serum biochemistry and hematological parameters were measured after the indicated treatments. Liver function–related markers: alanine aminotransferase (ALT) (a), aspartate aminotransferase (AST) (b), and alkaline phosphatase (ALP) (c). Renal function–related markers: blood urea nitrogen (BUN) (d) and creatinine (CREA) (e). Complete blood count (CBC) parameters: white blood cell count (WBC) (f), red blood cell count (RBC) (g), hemoglobin (HGB) (h), platelet count (PLT) (i), hematocrit (HCT) (j), mean corpuscular volume (MCV) (k), lymphocyte percentage (Lymph%) (l), and granulocyte percentage (Gran%) (m). (n) Body weight changes during the observation period. (o) HE‐stained sections of the heart, liver, spleen, lungs, kidneys and bladder of the mice with different treatments. Scale bars, 100 µm.

Whether AF/D can be a safe therapeutic approach for in vivo use is a critical issue that needs to be evaluated. To assess the biosafety of AF/D, comprehensive physiological and biochemical evaluations were conducted in mice. First, we examined the effects of AF/D on healthy mice following intravesical instillation. Eight‐week‐old healthy female C57BL/6 mice were used, and intravesical instillation was administered every 3 days for a total of four treatments. Blood, serum, weight and major organ samples were collected after treatment for analysis. Blood tests, as well as liver and kidney function indicators, including alanine aminotransferase (ALT), aspartate aminotransferase (AST), alkaline phosphatase (ALP), blood urea nitrogen (BUN), creatinine (CREA), direct bilirubin (DBIL), total bilirubin (TBIL), albumin (ALB), gamma‐glutamyl transferase (γ‐GT), total bile acid (TBA), uric acid (UA), and urea (UREA), were measured. The serum biochemistry results of the AF/D‐treated mice showed that all these parameters were within normal ranges, indicating no significant adverse effects on liver or kidney function (Figure 8a–e and Figure S21). Similarly, blood routine indicators such as white blood cell count (WBC), red blood cell count (RBC), and hemoglobin (HGB) were also within normal ranges (Figure 8f–m and Figure S22). Body weight was monitored throughout the study. Compared to the other groups, BCG‐treated mice showed significant weight loss (20.07%, Figure 8n). This magnitude of weight loss exceeds minor physiological fluctuation, suggesting an adverse effect on overall health status [66]. In contrast, mice in the AF/D group showed no significant body weight change during treatment and the post‐treatment observation period, with fluctuations of no more than 1.44%, supporting the favorable tolerability and biosafety of this therapy. Therefore, compared with BCG, AF/D not only promotes immunity but also shows improved biosafety. These results suggest that AF/D showed favorable tolerability in the current biosafety evaluation. Notably, under the tested conditions, BCG‐treated mice exhibited marked body weight loss, whereas AF/D‐treated mice showed minimal body weight fluctuation. However, this comparison was limited to biosafety assessment and does not indicate a direct therapeutic efficacy comparison between AF/D and BCG. It should be noted that BCG was included in this study only as a clinically relevant reference for biosafety evaluation, and a direct therapeutic efficacy comparison between AF/D and BCG was not performed.

Additionally, hematoxylin and eosin (H&E) staining of major organs (heart, liver, spleen, lung, kidney, and bladder) showed no significant pathological changes in the AF/D‐treated mice, and the tissue structures remained intact. Similarly, the AKK‐treated and AF‐treated groups did not exhibit notable toxicity, as their blood tests, serum biochemistry, weight changes, and histological analysis of major organs by H&E staining were comparable to those of the PBS‐treated group (Figure 8o). It should be noted that AF/D was not designed as an actively tumor‐targeted formulation, and exposure of normal urothelial cells to AF/D‐derived DOX cannot be completely excluded. Therefore, the present study does not demonstrate tumor‐selective cytotoxicity or complete avoidance of normal urothelial exposure. Nevertheless, repeated intravesical administration of AF/D to healthy mice caused no evident histopathological injury to the bladder, and the urothelial structure remained intact under the tested treatment regimen. Together with the absence of significant body weight loss, hematological abnormalities, or serum biochemical abnormalities, these findings support favorable local and systemic tolerability under the tested conditions. These results confirm that AF/D demonstrates good biosafety for in vivo applications.

## 3 Conclusion

In conclusion, we developed an intravesical bacteria‐based chemo‐immunotherapeutic platform (AF/D) that integrates pasteurized *Akkermansia muciniphila* (AKK), amine‐terminated F127, and doxorubicin (DOX). AF/D demonstrated high drug‐loading efficiency and pH‐responsive release behavior. AF/D outperformed conventional chemotherapy by strongly inhibiting bladder cancer cell proliferation, migration, and invasion while inducing profound cell death. This antitumor effect was associated with a coordinated cascade involving NF‐κB pathway suppression, DNA damage, mitochondrial dysfunction, ROS accumulation, glutathione depletion, lipid peroxidation, and GPX4 downregulation, which together contributed to the simultaneous activation of apoptosis and ferroptosis. Notably, AF/D also transformed tumor cell death into an immunogenic process, as evidenced by enhanced calreticulin exposure, HMGB1 release, and ATP secretion. These effects were accompanied by DCs maturation, M1‐like macrophage polarization, enhanced antigen cross‐presentation by DCs, and robust CD8^+^ T‐cell priming. In orthotopic bladder tumor models, intravesical AF/D achieved superior tumor suppression and prolonged survival, while remodeling both local and systemic antitumor immunity with a favorable safety profile. By integrating localized chemotherapy with bacteria‐mediated immunomodulation, AF/D establishes a mechanistically synergistic therapeutic paradigm that links multimodal tumor killing with durable immune activation. It should also be noted that AF/D was not designed as an active tumor‐targeting system, and the present study does not demonstrate specific recognition of bladder tumor cells over normal urothelial cells. Overall, this work highlights the potential of AF/D as a promising intravesical therapeutic strategy for bladder cancer and provides a versatile framework for the development of next‐generation chemo‐immunotherapies

## 4 Materials and Methods

### 4.1 Materials and Reagents

Pluronic F127 was supplied by Tanshtech (Guangzhou, China). N‐(3‐dimethylaminopropyl)‐N’‐ethylcarbodiimide hydrochloride (EDCI) was purchased from AbMole BioScience, Inc. (Houston, TX, USA). Doxorubicin (DOX) was purchased from Merck (Darmstadt, Germany). The Cell Counting Kit‐8 (CCK‐8) was obtained from ZETA Life (San Francisco, USA). RPMI‐1640, Dulbecco's modified Eagle's medium (DMEM), 0.25% trypsin‐EDTA, fetal bovine serum (FBS), and penicillin‐streptomycin were acquired from Gibco (Grand Island, USA). The YF 488‐Annexin V/RedNucleus II apoptosis kit was obtained from UElandy (Suzhou, China). Anti‐GPX4, and anti‐HMGB1 rabbit monoclonal antibodies were purchased from Abcam (Shanghai, China). Rabbit monoclonal primary antibodies against CRT and cleaved caspase‐9 used for immunofluorescence staining were purchased from Proteintech Group, Inc. (Wuhan, China). Antibodies against Bax, Bcl‐2, caspase‐9, and cleaved caspase‐9 were purchased from Cell Signaling Technology (Massachusetts, USA). DAPI was obtained from Beyotime (Shanghai, China). Fluorophore‐conjugated antibodies including FITC‐anti‐mouse CD80, APC‐anti‐mouse CD86, PE‐anti‐mouse F4/80, FITC‐anti‐mouse H‐2K^b^, PE‐anti‐mouse CD3, FITC‐anti‐mouse CD8, and APC‐anti‐mouse CD4 were purchased from BioLegend (San Diego, USA).

### 4.2 Cell Lines and Animals

Murine bladder cancer cell lines (MB49, MB49‐OVA, and MB49‐luciferase/MB49‐Luc) were maintained in high‐glucose DMEM containing 10% fetal bovine serum (FBS) and 100 U/mL penicillin‐streptomycin, and incubated at 37°C under humidified conditions with 5% CO_2_ 8‐week‐old female C57BL/6 mice were obtained from Zhiyuan Biotechnology Co., Ltd. (Guangzhou, China). All animal experiments were approved by and conducted under the supervision of the Institutional Animal Care and Use Committee of Shenzhen Following Precision Medical Research Institute, Luohu Hospital Group.

### 4.3 Synthesis of AF/D

AKK (OD = 1), amine‐terminated F127 (10 mg), and EDCI (4 mg) were dispersed in ultrapure water and stirred for 12 h to allow EDCI‐mediated amide bond formation between carboxyl groups on the AKK surface and terminal amine groups of F127. Doxorubicin (DOX, 500 µg) was then added, followed by an additional 6 h of stirring. The mixture was subsequently centrifuged at 12 000 rpm for 10 min and washed twice to remove unbound components. The final pellet was collected as AF/D.

### 4.4 Cytotoxicity Assay

Cell viability was quantified using a CCK‐8 assay. MB49 cells were seeded in 96‐well plates at a density of 1 × 10^4^ cells per well and exposed to AKK, DOX, or AF/D for 24 h. Subsequently, CCK‐8 solution was added and the plates were incubated at 37°C for 1 h. Absorbance was then recorded at 450 nm.

### 4.5 Cellular Uptake Analysis

MB49 cells (8 × 10^4^ cells/mL) were plated on glass coverslips in 24‐well plates and allowed to adhere for 12 h, after which they were incubated with AF/D for 1, 4, or 7 h. Cells were then fixed with 4% paraformaldehyde and stained with Actin‐Tracker Green‐488 to visualize the cytoskeleton. Fluorescence images were acquired using a confocal laser scanning microscope (CLSM; LSM‐800, Carl Zeiss, Germany). For flow cytometry, MB49 cells (3 × 10^5^ cells/well) were seeded in 12‐well plates and subjected to the same treatment conditions prior to FCM analysis (Shenzhen Weigong Bio Co., Ltd.).

### 4.6 Cell Clonogenic Assay

MB49 cells were plated in 6‐well plates at 2000 cells per well and allowed to attach for 12 h. The cultures were then exposed to PBS, AKK, AF, DOX, or AF/D and maintained for 10 days to permit colony formation. After colonies became visible, cells were fixed with 4% paraformaldehyde and stained with 0.5% crystal violet. Colony images were subsequently recorded using a digital camera.

### 4.7 Wound Healing Assay

MB49 cells were plated in 6‐well plates and grown to 80%–90% confluence. A linear scratch was introduced across the cell monolayer using a sterile 200‐µL pipette tip to create an artificial wound. The cells were then incubated with PBS, AKK, AF, DOX, or AF/D for 24 h. Images of the wound were acquired by optical microscopy at 0 h and after 24 h of incubation. The wound area was quantified using ImageJ, and the percentage of wound closure was calculated to determine the migratory capacity.

### 4.8 Transwell Invasion Assay

Transwell invasion assays were performed using Transwell inserts pre‐coated with Matrigel. Matrigel was diluted 1:8 in serum‐free DMEM and added to the upper surface of the insert membrane, followed by incubation at 37°C to allow gel formation. MB49 cells (1 × 10^5^) were resuspended in 200 µL serum‐free DMEM and seeded into the upper chamber of a Transwell insert. The lower chamber was filled with 1000 µL DMEM supplemented with 10% fetal bovine serum (FBS) to serve as a chemoattractant. PBS, AKK, AF, DOX, or AF/D was added to the upper chamber, and the cells were allowed to invadefor 24 h. After incubation, cells remaining on the upper surface of the membrane were gently removed with a cotton swab, and the inserts were rinsed three times with PBS. Cells that invaded to the underside of the membrane were fixed with 4% paraformaldehyde for 20 min and stained with 0.1% crystal violet for 10 min. Images were captured under an optical microscope, and invaded cells were quantified by counting five randomly selected fields per insert using ImageJ.

### 4.9 Live/Dead Cell Staining Assay

MB49 cells were seeded at a density of 8 × 10^5^ cells per dish in 3.5 cm cell culture dishes. After 24 h of treatment with PBS, AKK, AF, DOX, or AF/D, the medium was removed, and the cells were washed with cold PBS and stained with a Calcein‐AM/PI cell viability kit (Beyotime). Images were then captured using CLSM.

### 4.10 Detection of DNA Damage Marker γ‐H_2_AX

MB49 cells were plated onto glass coverslips in 24‐well plates at a density of 1 × 10^6^ cells per well and allowed to attach for 12 h. The medium was then replaced, and the cells were incubated with PBS, AKK, AF, DOX, or AF/D for 24 h. After treatment, cells were rinsed with PBS and stained with an anti‐γ‐H_2_AX primary antibody at 37°C for 2 h, followed by incubation with an Alexa Fluor 488‐conjugated secondary antibody for 1 h. Fluorescence images were acquired using CLSM.

### 4.11 Apoptosis Analysis

MB49 cells (3 × 10^5^ cells/well) were subjected to the treatments described above, stained using the YF 488‐Annexin V/RedNucleus II apoptosis detection kit (UElandy, Suzhou, China), and subsequently analyzed by FCM.

### 4.12 Western Blotting Analysis

After exposure to the indicated treatments, MB49 cells (1 × 10^7^ cells per well) were lysed in RIPA buffer supplemented with protease and phosphatase inhibitors to extract total proteins. Protein concentrations were determined using a BCA assay. Equal amounts of protein were resolved by SDS–PAGE and transferred onto PVDF membranes. The membranes were then incubated with primary antibodies, followed by HRP‐conjugated secondary antibodies, and protein bands were visualized using an enhanced chemiluminescence (ECL) substrate.

### 4.13 Cellular Immunofluorescence

MB49 cells were plated on glass coverslips. Following the designated treatments, cells were fixed, permeabilized, and blocked, after which immunofluorescence staining was performed to detect the proteins of interest. Fluorescence images were collected using confocal laser scanning microscopy (CLSM), and quantification was carried out under identical exposure parameters across all groups.

### 4.14 ROS, GSH/GSSG, and LPO Detection

Intracellular ROS, GSH/GSSG, and lipid peroxidation (LPO) were evaluated in MB49 cells using standard assays. For ROS measurement, MB49 cells (3 × 10^5^ cells/well) were plated in 12‐well plates, exposed to the indicated treatments for 24 h, and then incubated with DCFH‐DA for 20 min prior to analysis by FCM. For determination of the GSH/GSSG ratio, MB49 cells (1 × 10^6^ cells/well) were seeded in 6‐well plates, lysed after treatment, and the GSH/GSSG ratio was quantified using a microplate reader. To assess LPO, MB49 cells (1 × 10^6^ cells/well) were cultured in 6‐well plates, stained with BODIPY 581/591 C11 for 30 min, and lipid peroxidation was analyzed by FCM.

### 4.15 Mitochondrial Membrane Potential Detection

Mitochondrial membrane potential (ΔΨm) was assessed in MB49 cells. Cells (1 × 10^6^ cells/well) were plated in 6‐well plates and, following the indicated treatments, stained with Rhodamine 123 for 30 min. Fluorescence signals were subsequently analyzed by FCM and CLSM.

### 4.16 In Vitro DC Maturation

Bone marrow‐derived dendritic cells (BMDCs) were generated from bone marrow harvested from 4‐week‐old C57BL/6 mice and differentiated in the presence of granulocyte–macrophage colony‐stimulating factor (GM‐CSF, 20 ng/mL) and interleukin‐4 (IL‐4, 10 ng/mL) for 5 days. The resulting BMDCs were then incubated with the indicated formulations for 24 h, stained with CD11c‐PE, CD80‐FITC, and CD86‐APC, and subsequently analyzed by FCM.

### 4.17 Evaluation of BMDC Maturation Induced by Tumor‐Cell‐Conditioned Medium

MB49 cells were treated with PBS, AKK, AF, DOX, or AF/D for 24 h. The culture supernatants were subsequently collected and centrifuged to remove cells and cellular debris. The resulting conditioned media were transferred to BMDC cultures and incubated. BMDCs were then collected and stained with antibodies against CD11c, CD80, and CD86. The proportion of CD80^+^CD86^+^ mature BMDCs was determined by flow cytometry.

### 4.18 Detection of DNA Damage Marker γ‐H_2_AX

MB49 cells were plated onto glass coverslips in 24‐well plates at a density of 1 × 10^5^ cells per well and allowed to attach for 12 h. The medium was then replaced, and the cells were incubated with PBS, AKK, AF, DOX, or AF/D for 24 h. After treatment, cells were rinsed with PBS and stained with an anti‐γ‐H_2_AX primary antibody at 37°C for 2 h, followed by incubation with an Alexa Fluor 488‐conjugated secondary antibody for 1 h. Fluorescence images were acquired using CLSM.

### 4.19 In Vitro Macrophage Polarization

RAW264.7 cells (1 × 10^6^ cells/well) were plated in 6‐well plates and exposed to the indicated formulations. Cells were then stained with F4/80‐PE and CD80‐FITC, and macrophage polarization was evaluated by FCM.

### 4.20 Evaluation of Immunogenic Cell Death (ICD) In Vitro

Following exposure of MB49 cells to the indicated formulations for 24 h, culture supernatants were harvested and extracellular ATP was quantified using a luminescence‐based ATP assay to evaluate treatment‐induced immunogenic cell death (ICD). In parallel, the treated cells were processed for immunofluorescence staining, and confocal laser scanning microscopy was used to assess calreticulin (CRT) surface exposure and the nuclear‐to‐cytoplasmic redistribution of high‐mobility group box 1 (HMGB1), thereby further verifying the activation of ICD‐associated signals.

### 4.21 Establishment of the Orthotopic MB49‐Luc Bladder Cancer Model

MB49‐Luciferase cells (5 × 10^6^) were suspended in 20 µL sterile 1× PBS. After the mice were fully anesthetized, the bladder was surgically exposed, and the cell suspension was injected into the bladder wall. Successful injection was indicated by the appearance of a clear, disc‐like bulge at the inoculation site. The abdominal incision was subsequently closed, and the vital signs of the mice were monitored. Tumor establishment was verified by in vivo bioluminescence imaging on day 3 after inoculation.

### 4.22 In Vivo Antitumor Efficacy and Immune Activation

After tumor establishment, mice were randomly assigned to five groups and treated with PBS, AKK, AF, DOX, or AF/D via transurethral intravesical instillation according to the dosing schedule shown in Figure 7a. Briefly, mice were anesthetized, and the flexible tube from a sterile disposable intravenous indwelling catheter was gently inserted into the bladder through the urethra. After emptying the bladder, the corresponding formulation was instilled intravesically and retained in the bladder for 2 h. Tumor progression was longitudinally tracked by bioluminescence imaging. Following completion of the treatment regimen, mice were sacrificed, and bladders were harvested. The spleen and bladder tissues were then collected and processed for flow cytometric analysis to evaluate dendritic cell maturation, M1 macrophage polarization, and CD4^+^/CD8^+^ T‐cell subsets.

### 4.23 Statistical Analysis

Comparisons between two groups were performed using an unpaired Student's *t*‐test, while differences among three or more groups were assessed by one‐way analysis of variance (ANOVA). A *p*‐value < 0.05 was considered statistically significant. Significance levels are indicated as ^*^ *p* < 0.05, ^**^ *p* < 0.01, ^***^ *p* < 0.001, and ^****^ *p* < 0.0001.

## Funding

This work was supported by Shenzhen Medical Research Fund (A2502022, A2503070); The National Natural Science Foundation of China (82572292, 92359202); Sanming Project of Medicine in Shenzhen (SZSM202411033, SZSM202211009); Shenzhen Science and Technology Innovation Commission (RCJC20200714114557005, JCYJ20240813144023031); National Key Research and Development Program of China (2025YFF0515300, 2025YFF0515304); Shenzhen Science and Technology Program (SYSRD20250529113406008, JCYJ20230807141009020, QNXMC20250701091517023, QNXMC20250701091514019); Shenzhen Engineering Research Center (XMHT20220104016); Project funded by China Postdoctoral Science Foundation (2024M762130).

## Ethics Statement

All procedures involving animals were carried out in full compliance with the applicable guidelines and regulations for the care and use of laboratory animals. The study protocol received formal approval from both the Experimental Committee and the Ethics Committee of Shenzhen Following Precision Medical Research Institute, Luohu Hospital Group (Approval No.AP‐SZZX‐2025‐11‐002).

## Conflicts of Interest

The authors declare no conflicts of interest.

## Supporting information




**Supporting File 1**: advs77241‐sup‐0001‐SuppMat.docx.

**Supporting File 2**: advs77241‐sup‐0002‐SuppMat.docx.

**Supporting File 3**: advs77241‐sup‐0003‐S1.xlsx.

## Data Availability

The data that support the findings of this study are available from the corresponding author upon reasonable request.
