# Supplementary material for: An Intravesical Akkermansia muciniphila‐Based Chemo‐Immunotherapeutic Platform for Bladder Cancer
Source: Adv Sci (Weinh). 2026 Aug 17;13(62):e77241. doi: 10.1002/advs.77241 (PMC13479586; doi:10.1002/advs.77241)
Supplement: Supplementary file 1 [file ADVS-13-e77241-s002.docx]

Supporting Information 2

**Supporting Information 2** Original western blot images for Bax, Bcl-2, GPX4, Caspase-9, Cleaved Caspase-9 and Tubulin.
