# Supplementary material for: An Intravesical Akkermansia muciniphila‐Based Chemo‐Immunotherapeutic Platform for Bladder Cancer
Source: Adv Sci (Weinh). 2026 Aug 17:e77241. Online ahead of print. doi: 10.1002/advs.77241 (PMC13479586; doi:10.1002/advs.77241)
Supplement: Supplementary file 2 [file ADVS-9999-e77241-s003.docx]

Supporting Information

**An Intravesical *Akkermansia muciniphila*-Based Chemo-Immunotherapeutic Platform for Bladder Cancer**

Rongkang Li ^1,2, #^, Anguo Zhao ^2,3, #^, Qi Zhuang ^2,4, #, *^, Lei Peng ^2^, Rui Liang ^2^, Jiadong Zhao ^2^, Yang Liu ^1,2^, Dashi Deng ^2^, Peng Yu ^1,2^, Xiqi Peng ^2^, Shaohua Zhang ^2, *^, Song Wu ^1,2, *^

^1^ Department of Urology, The Second Hospital & Clinical Medical School, Lanzhou University, Lanzhou 730030, China.

^2^ Department of Urology, South China Hospital, Medical School, Shenzhen University, Shenzhen, 518116, China.

^3^ Department of Urology, The Fourth Affiliated Hospital of Soochow University, Medical Center of Soochow University, Suzhou Dushu Lake Hospital, Suzhou 215000 China.

^4^ Guangdong Key Laboratory for Biomedical Measurements and Ultrasound Imaging, National-Regional Key Technology Engineering Laboratory for Medical Ultrasound, School of Biomedical Engineering, Shenzhen University Medical School, Shenzhen, 518116 China.

^#^ Both authors contributed equally to this work

* Corresponding author: Song Wu. E mail: wusonglab@outlook.com;

* Corresponding author: Shaohua Zhang. E‑mail: zhangshaohua@szu.edu.cn;

* Corresponding author: Qi Zhuang. E‑mail: qizhuang@szu.edu.cn;

Funding: This work was supported by Shenzhen Medical Research Fund (A2502022, A2503070); The National Natural Science Foundation of China (82572292, 92359202); Sanming Project of Medicine in Shenzhen (SZSM202411033, SZSM202211009 ); Shenzhen Science and Technology Innovation Commission (RCJC20200714114557005, JCYJ20240813144023031); National Key Research and Development Program of China (2025YFF0515300, 2025YFF0515304); Shenzhen Science and Technology Program (SYSRD20250529113406008, JCYJ20230807141009020, QNXMC20250701091517023, QNXMC20250701091514019); Shenzhen Engineering Research Center (XMHT20220104016); Project funded by China Postdoctoral Science Foundation (2024M762130).

**Supplementary Figure 1** Stability evaluation of AF/D in buffer. Hydrodynamic size distribution of AF/D after incubation in buffer for 0, 24, 48, and 72 h, as measured by dynamic light scattering.

**Supplementary Figure 2** Representative low-magnification TEM images of AKK, AF, and AF/D showing multiple particles in a broader field of view. Scale bars: 2 μm for AKK and AF; 1 μm for AF/D.

**Supplementary Figure 3** UV-Vis absorption spectra of DOX, and AF/D.

**Supplementary Figure 4** Drug loading efficiency of DOX in A/D and AF/D. A/D was prepared by direct adsorption of DOX onto AKK without amine-terminated F127 modification, while AF/D was prepared using amine-terminated F127-modified AKK. Data are shown as mean ± SD, n = 3.

**Supplementary Figure 5** MB49 cells were treated with free DOX or AF/D at the indicated DOX concentrations, and the relative intracellular DOX fluorescence intensity was quantified by flow cytometry (FCM).

**Supplementary Figure 6** Cell viability of MB49 cells treated with varying concentrations of AKK measured by CCK-8 assay. The slight value above 100% in the low-dose AKK group should be interpreted as minor variation in the CCK-8 metabolic activity-based readout rather than evidence of AKK-induced tumor cell proliferation.

**Supplementary Figure 7** GO analysis of genes differentially expressed between PBS- and AF/D-treated MB49 cells.

**Supplementary Figure 8** Validation of western blot reproducibility for Bax, Bcl-2, and Tubulin. Band images from two additional independent western blot replicates showing Bax, Bcl-2, and Tubulin expression in MB49 cells after treatment with PBS, AKK, AF, DOX, or AF/D. These data, together with the representative blot shown in the main figure, support the quantitative analysis based on three independent experiments.

**Supplementary Figure 9** Western blot analysis of cleaved caspase-9 (CC9) expression in MB49 cells, with tubulin as the loading control. Densitometric quantification of cleaved caspase-9 (CC9) levels based on grayscale analysis.

**Supplementary Figure 10** FCM profiles of intracellular lipid peroxidation levels in MB49 cells after different treatments.

**Supplementary Figure 11** FCM analysis were performed to assess treatment-induced alterations in mitochondrial membrane potential in MB49 cells.

**Supplementary Figure 12** CLSM image showing changes in mitochondrial membrane potential in MB49 cells. Scale bars, 40μm.

**Supplementary Figure 13** FCM analysis of M1 macrophage polarization in RAW264.7 cells under different treatments.

**Supplementary Figure 14** BMDC maturation induced by conditioned media from treated MB49 cells. MB49 cells were treated with PBS, AKK, AF, DOX, or AF/D for 24 h. The culture supernatants were collected by centrifugation and subsequently incubated with BMDCs. Representative flow cytometry plots and quantitative analysis of CD80⁺CD86⁺ mature BMDCs are shown.

**Supplementary Figure 15** Urinary clearance of DOX after intravesical AF/D administration. DOX concentration in urine collected at different time points after intravesical administration of AF/D, as quantified by HPLC. AF/D containing DOX at 1 mg/mL was administered at a volume of 50 μL, followed by urethral clamping for 2 h. The time point immediately after releasing the clamp was defined as 0 h. Data are shown as mean ± SD, n = 3. The y-axis is shown on a logarithmic scale because of the wide concentration range.

**Supplementary Figure 16** Intravesical retention of Cy5.5-labeled A/D and AF/D after perfusion. In vivo fluorescence imaging of mice after intravesical administration of Cy5.5-labeled A/D or AF/D at 0, 4, 8, 12, 24, and 48 h after release of urethral clamping. The time point immediately after releasing the clamp was defined as 0 h.

**Supplementary Figure 17** Time-dependent distribution of DOX in bladder tumor tissues after intravesical AF/D administration. Representative fluorescence images of bladder tumor sections collected at 0, 0.5, 1, 2, 4, and 8 h after release of urethral clamping. The time point immediately after releasing the clamp was defined as 0 h. Blue, DAPI; red, DOX.

**Supplementary Figure 18** FCM analysis of DC activation in the bladder tumors of mice subjected to different treatments.

**Supplementary Figure 19** FCM images showing M1 macrophage in the bladder tumors of mice subjected to different treatments.

**Supplementary Figure 20** Statistical analysis of M1 macrophage in the bladder tumors of mice treated with different groups.

**Supplementary Figure 21** FCM images showing CD4^+^T cells (a) and CD8^+^T cells (b) in the bladder tumors of mice subjected to different treatments.

**Supplementary Figure 22** Additional serum biochemistry and hematology parameters for biosafety evaluation. Additional liver/kidney function–related serum indices and hematological parameters that were not included in the figure 6 are shown for mice receiving intravesical instillation of PBS, AKK, AF, DOX, or AF/D. Serum markers include direct bilirubin (DBIL), total bilirubin (TBIL), albumin (ALB), gamma-glutamyl transferase (γ-GT), total bile acid (TBA), uric acid (UA), and urea (UREA). Hematological parameters include lymphocyte count (Lymph#), monocyte count (Mon#), granulocyte count (Gran#), monocyte percentage (Mon%), mean corpuscular hemoglobin (MCH), mean corpuscular hemoglobin concentration (MCHC), red cell distribution width (RDW), mean platelet volume (MPV), platelet distribution width (PDW), and plateletcrit (PCT).
